# Fisheye-Based Method for GPS Localization Improvement in Unknown Semi-Obstructed Areas

**DOI:** 10.3390/s17010119

**Published:** 2017-01-17

**Authors:** Julien Moreau, Sébastien Ambellouis, Yassine Ruichek

**Affiliations:** 1 UTBM IRTES-SET, Rue Ernest Thierry Mieg, 90010 Belfort CEDEX, France; yassine.ruichek@utbm.fr; 2 IFSTTAR-COSYS-LEOST, 20 rue Élysée Reclus, BP 70317 59666 Villeneuve d’Ascq CEDEX, France; sebastien.ambellouis@ifsttar.fr

**Keywords:** vehicle positioning, GPS, fisheye stereovision, 3D point cloud

## Abstract

A precise GNSS (Global Navigation Satellite System) localization is vital for autonomous road vehicles, especially in cluttered or urban environments where satellites are occluded, preventing accurate positioning. We propose to fuse GPS (Global Positioning System) data with fisheye stereovision to face this problem independently to additional data, possibly outdated, unavailable, and needing correlation with reality. Our stereoscope is sky-facing with 360° × 180° fisheye cameras to observe surrounding obstacles. We propose a 3D modelling and plane extraction through following steps: stereoscope self-calibration for decalibration robustness, stereo matching considering neighbours epipolar curves to compute 3D, and robust plane fitting based on generated cartography and Hough transform. We use these 3D data with GPS raw data to estimate NLOS (Non Line Of Sight) reflected signals pseudorange delay. We exploit extracted planes to build a visibility mask for NLOS detection. A simplified 3D canyon model allows to compute reflections pseudorange delays. In the end, GPS positioning is computed considering corrected pseudoranges. With experimentations on real fixed scenes, we show generated 3D models reaching metric accuracy and improvement of horizontal GPS positioning accuracy by more than 50%. The proposed procedure is effective, and the proposed NLOS detection outperforms CN0-based methods (Carrier-to-receiver Noise density).

## 1. Introduction

It is a challenging problem to correct PR (PseudoRange) delays in order to improve GNSS (Global Navigation Satellite System) localization. PR are one of the raw data used to compute a GNSS position on the terrestrial globe. They represent the measured distance between the receiver and satellites, that means they typically include bias. A pseudorange is usually around some tens of thousands of kilometres, but an estimation error as small as some metres brings to an inaccurate positioning that can easily reach tens of metres error.

The presented procedure deals with local PR delay in an autonomous way. It does not rely on embedded data, which has big advantages, especially the removal of the positioning problem in a recorded 3D model. The idea is to generate a 3D local model on line, with a sky-facing fisheye binocular stereovision configuration allowing a 360° × 180° field of view in order to see all around the vehicle. The main interest of such a 3D reconstruction, centred on a local vehicle frame, is that the GNSS antenna position is naturally known with high accuracy inside the model. This vision-based method is chosen because of its aptitudes to easily be integrated on a vehicle, to get dense data about surrounding area with only one acquisition (two pictures), and its low-cost, compared to laser sensors. For now the procedure is not real-time, our aim is to show its feasibility and efficiency.

### 1.1. Principle of GNSS Localization

GNSS position solving is explained in [1] (Chapter 6). Geometrically, it is possible to compute a 2D position by trilateration thanks to the knowledge of at least two other different reference positions and the range between these positions and the position to be computed. Each ranging measurement give a circle centred on the reference position, and both circles intersects at two points. For most applications, the ambiguity can be solved because only one solution is viable. Otherwise a supplementary reference position and linked ranging measurement are necessary. The same applies for a 3D position, which needs at least three other different positions and corresponding distances, and so on, it is possible to estimate a vector of *X* unknowns if we know *X* other vectors in the same frame and corresponding ranges.

To perform the geo-localization, a receiver knows the positions of every constellation’s satellite and computes their range to its antenna. These computed distances are the PR. A PR is computed from speed of light and the time the satellite signal takes to reach receiver’s antenna. Assuming synchronised satellites and receiver clocks, signal route time is equal to the difference between time of arrival and time of emission.

In practice, however, satellites and receiver clocks are not synchronised. This results in over or underestimated ranges measurements. Satellites clock offsets are permanently reviewed by control stations, and thus, offsets compared to an atomic clock are included in transmitted navigation messages for the receiver. The only remaining unknown is the receiver clock offset from system time. So, the receiver time error can be processed as a fourth parameter to estimate, and GNSS navigation solution is computed this way in a 4D frame: (X,Y,Z,t) containing 3D coordinates and receiver’s time synchronisation. That is, to estimate GNSS 3D position, at least 4 satellites are needed.

In open and uniform area a pseudorange fits well with the real distance. The reality is different, pseudorange propagation is perturbed by the environment:

1. a signal is delayed in different Earth’s atmosphere layers (especially the ionosphere and troposphere) [1] (pp. 247–250);
2. in semi-obstructed area, a signal may be received after multiple reflections on the structures, and even worse, it may be received several times by multipath effect [1] (pp. 254–257).

The first point is well corrected by DGNSS (Differential GNSS) processes [1] (pp. 279–283) or the simultaneous use of signals of multiple frequencies. The second point is more difficult to correct as it depends on the local reception area. Multipaths can be mitigated by antennas and receivers designs, mapping techniques and filtering in the navigation processor [1] (pp. 294–296), but local reflections cause pseudorange delays.

For presented experiments, we use the GPS (Global Positioning System) solution. This is the most used GNSS, as it is historically the first available and receiving devices are common. GPS positioning is given in the WGS84 frame (World Geodetic System 1984).

The aim of this paper is to present an autonomous procedure to compute these pseudorange delays in order to increase GPS localization accuracy.

### 1.2. Multipath Effect

**Figure 1:**
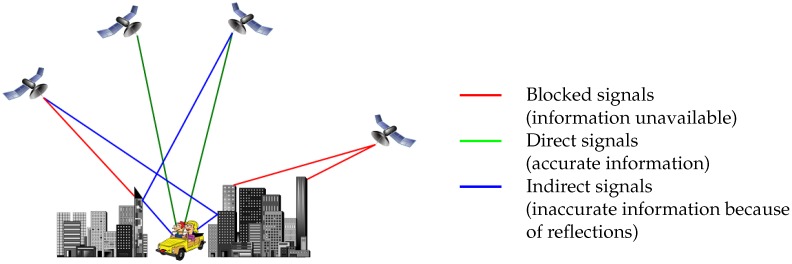
Multipath effect due to rays reflections.

The Figure 1 illustrates multipath effect. Best data to get are from direct signals, in this case GNSS signal reception is designated LOS (Line Of Sight). When the reception does not occur via a direct path but only via a reflected path (no direct signal of that particular satellite), it is designated NLOS (Non Line Of Sight). It is shown that a signal can be received several times via different ways, this is the multipath effect.

The presented research focuses on NLOS reception and not on multipath effect. Only NLOS signals can be delayed by local structures. So, the first step is to identify NLOS satellites. Then, a delay estimation is proposed in order to correct NLOS pseudoranges and improve GPS positioning.

In the following, we present research works related to our proposed framework. Table 1 summarizes the related topics and their state of the art references.

### 1.3. State-of-the-Art of NLOS Correction Methods

Authors of [18] propose a study of GNSS signal propagation and local interferences. In reality signal propagation forms a cone becoming larger with the range. The maximum power is at its centre. Reflecting surfaces reflect and disperse the signal with various parameters. Ercek et al. simulate data in an urban canyon, a straight street with buildings of different heights. In order to simplify the problem, the reception with the strongest power only is usually taken into account and considered as a punctual geometric ray. Ercek et al. formulate probabilities for a received signal to be NLOS and to present a reflection or a diffraction, according to the area geometric configuration and the power of the signal.

Considering the punctual geometric ray for the signal propagation, many NLOS analysis methods have been studied to improve GNSS localization accuracy. We can sort bibliography within these criteria:

- Authors aiming to detect NLOS signals without correcting pseudorange delays:
  - Most of the time they only exclude NLOS signals for position computation;
  - Some of them use the NLOS information inside specific procedures.
- Authors trying to correct pseudorange delays;
- And authors applying a different strategy, consisting in testing and validating many positions in a 3D model of the area.

#### 1.3.1. NLOS Signals Detection and Exclusion

Some authors show that ignoring NLOS satellites can improve positioning accuracy. Therefore, excluding satellites increases the risks of a bad geometrical repartition of considered satellites, represented by the DOP (Dilution Of Precision), and so of a less accurate position estimation. In addition, if not enough satellites are taken for the localization computation, the service becomes unavailable. This procedure is efficient if more than 4 satellites are considered. This is the case for the following authors.

In [12], Peyret et al. detect NLOS satellites by generating a virtual view from the vehicle position in a recorded 3D model. Satellites are projected into this view. Structures act as a mask to identify NLOS satellites and exclude them.

Authors of [4] compare two methods of NLOS satellites identification: a vision-based method, segmentation of structures visible around the vehicle in roof-mounted fisheye images, described in [35], and a CN0 (Carrier-to-receiver Noise density) threshold, manually chosen according measurements. Marais et al. get better results with the second and easiest approach. By excluding NLOS signals with the CN0 threshold, they are able to decrease the localization inaccuracy in a sequence from 13 m to 4.6 m.

In [9], Obst et al. use 3D data of the environment from cartography and elevation maps in order to identify NLOS signals by ray-tracing. Positioning the receiver in the 3D model is needed. By excluding NLOS signals, for a sequence, they reduce the GNSS position error from 14 m to 5.2 m.

In [16], Kumar et al. do not use recorded 3D data. They build a 3D model with a lidar and use the scanned structures as a mask for NLOS satellites. For fixed positions, excluding NLOS signals allows an accuracy less than 1 m.

#### 1.3.2. NLOS Signals Filtering

Instead of exclusion, NLOS data can be treated anyway with a specific method according to their credibility.

- Smoothing using NLOS information:
  Authors of [10,11] use a recorded 3D model to detect NLOS satellites. They use a Kalman filter to smooth the localization estimation, taking into account LOS or NLOS status of the signals.
- Weighting of NLOS data:
  In [5], Tay and Marais weight the contribution of NLOS signals in the position computation according to the CN0. NLOS satellites are identified using the fisheye vision-based method described in [35]. Results seem better than a simple NLOS exclusion, in terms of accuracy and without loss of availability. For presented sequence, position error is reduced from 15 m to approximately 4 m.
- Shadow-matching:
  This method is used in [13]. The principle consists in testing a grid of candidates positions around the initial GNSS position that is computed. For each satellite, the aim is to use the 3D model and ray-tracing to describe areas where the satellite might be LOS, NLOS or totally blocked. The test is done by correlating the state of each satellite in the model with CN0 measurements of the reality. The candidate position with the best score is considered as the final estimated position.

#### 1.3.3. NLOS Pseudoranges Correction

NLOS PR correction does not affect the position computation principle : every PR is used in a standard way, they are adjusted to be improved according to propagation geometrical constraints. This is a direct localization rectification. Authors try to estimate the delay according to a single point projection model and geometric properties.

In [17] Marais et al. propose an extremely simplified model for pseudorange delay estimation. It considers one reflection, and takes into account only two parameters: satellite elevation and the orthogonal distance between the receiver and the reflecting wall. Authors of [3,18] present the same approach but more accurately by taking into account also the receiver’s azimuth.

In [3], Viandier proposes a more sophisticated method for estimating pseudorange delays. He presents a pseudorange noise model based on gaussian mixtures and Dirichlet process in order to correct them. Parameters are estimated by particular filter. Viandier considers following noise sources:

- multipath effect (a signal is received more than once)
- signal reflection (weaker signal)
- signal diffraction and diffusion (signal very weak, occurs when it passes through vegetation or reaches an edge)

Using this method, Viandier is able to improve the localization accuracy from 21 m to 11 m.

Authors of [19] propose a reflection model allowing one, two, or three and more reflections. This model supposes a street like a straight line with parallel opposite walls, and is defined with four parameters: the width, left and right heights, and the transversal position of the receiver. In their experiments, parameters are pre-recorded in the system. They use them to generate visibility mask and to determine the number of reflections in the case of an NLOS signal. With this pseudorange delay correction, they improve the position accuracy from 30% to 70% with real data.

Some authors like [14,15] try to compute the pseudorange delay thanks to a 3D map and a ray-tracing simulation method for a grid of candidate positions. By excluding impossible positions, they estimate the delay. Likelihood between real measures and theoretical estimations at the candidates positions is used to weight the final PR delay estimation.

In order to add a robust integrity measure, authors of [15] cumulate this method with RAIM FDE (Receiver Autonomous Integrity Monitoring, Fault Detection and Exclusion). This procedure excludes signals with a big difference between the theoretical pseudorange and the real measure.

To apply their methods, these authors use a recorded 3D model. Both their experiments are done for a smartphone receiver handled by a pedestrian. In [14], Miura et al. increase the accuracy from more than 34 m to 20 m, and in [15], Hsu et al. increase the accuracy from more than 20 m to 4 m.

### 1.4. NLOS Detection Methods Based on Vision

Recently, some authors have developed fisheye vision method to detect NLOS satellites. Their approach are used and validated through a NLOS pseudoranges exclusion or correction based on statistical models. A difficulty for the evaluation is that they often add dead-reckoning by GNSS and IMU (Inertial Measurement Unit) data fusion and an EKF (Extended Kalman Filter). This is the case in [6,8]. But, what is interesting are the improvements of raw GNSS positions, without any smoothing. Moreover, every implementation of EKF can have different parameters, so in this case no comparison is meaningful.

Authors of [6] present a fisheye vision-based method to detect NLOS satellites. They segment the colour image in order to detect sky and structures areas. This method is based on meanshift segmentation, which gives superpixels with well-detected boundaries. To distinguish sky and non-sky superpixels, Suzuki et al. measure a theoretic CN0 for direct signals in open-sky environment. Each satellite validating a signal according to this model is LOS with high reliability, that means the superpixel where it is projected corresponds to the sky. Colour histogram of each superpixel is compared to sky superpixels detected this way. Those that are close to sky characteristics are recognized as sky superpixels. NLOS satellite detection is done by looking in which superpixel projects each satellite in the fisheye image, according to the label given to the superpixel. In addition, they propose a fisheye vision-based method to know the vehicle orientation. Orientation is necessary to correctly project GNSS satellites in the fisheye image. They apply a strategy of NLOS satellites rejection for the final localization.

In [8], Sanroma et al. use a grey levels sky-facing fisheye camera in order to detect NLOS satellites. Their method is based on Canny edge segmentation. They apply pre and post-processing in order to close the shapes. A floodfill operation is used to label the LOS area (sky). This needs the assumption of a LOS satellite based on a high probability CN0 threshold of 45 dBHz. To distinguish a NLOS satellite, they fuse CN0 thresholds with the label of the projection area containing the satellite in the fisheye image. They apply a pseudorange delay estimator based on measures in dense urban environment.

Authors of [7] evaluate segmentation methods for detecting sky and non-sky areas in sky-facing images. They use a colour ultra wide-angle camera with a 90° field of view. The idea is the same: projecting satellites in the images in order to detect NLOS ones according to the position in the picture. Evaluated image segmentation methods are: Otsu automatic threshold, Meanshift, HMRF-EM (Hidden Markov Random Fields Expectation-Maximization), and graph-cut. Post-processing are done in order to get two smoothed categories. They find that with no other specific processes, Otsu segmentation is the best both in time and accuracy. They apply a strategy of NLOS satellites rejection for the final localization. Their evaluation seems impartial as it is based on raw GNSS data. They use a measure of the pseudorange error residuals. RMS (Root Mean Square) of error residuals are decreased from 89.4 m to 19.5 m, using GPS position before and after detected NLOS rejection.

### 1.5. Bibliography on Calibration

GNSS localization is based on the PR computation between satellites and receiver. NLOS satellites are those that are not in the direct line of sight of the receiver, they are received following other ways. NLOS satellites distances are often seen longer than they truly are. It causes inaccuracy of the position estimation.

Several methods have been developed, based on local structures contained in recorded data. However, currently, no one is able to deal with the pseudoranges delay problem in unknown areas, and they imply a big difficulty to work: the receiver must be well positioned in the 3D data according to the true position in the real world.

Proposed approach to go through these problems consists in building the 3D model directly from the vehicle that is geo-localizing. In order to compute NLOS PR delays, the 3D model must be true-scale. 3D points of the scene can be computed by the use of the stereovision principle, as the human visual system to perceive depth. We use a fisheye stereovision setup to see all around the vehicle. The baseline between two views must be large (metre scale) to be able to build large-scale environments such as urban areas. Several authors computes 3D models from vision, and the procedure is divided in two parts:

- Cameras calibration
- Matching for 3D scene reconstruction

Calibration is necessary as it allows to match efficiently the points between two views according to geometric “epipolar” constraints.

#### 1.5.1. Pattern-Based Calibration

To calibrate means to estimate the relationship between points in the real world and their projections in the pictures. Generally, authors apply a parametric calibration, and assume that the projection is following a mathematical model [22]. Calibration parameters are estimated by minimizing the error between real points projections in the image and their projections according to the considered camera model. For stereovision, when cameras parameters are estimated, we can compute relationship between two or more rigidly linked cameras, allowing the computation of 3D points of the scene.

Most of the calibration methods are manual and based on a pattern. A pattern is a grid where points positions are accurately known in 3D. In order to proceed the calibration, the user takes enough pictures with different pattern positions relatively to the camera, to cover the maximum image surface. Because pattern dimensions are known in the real world, this kind of calibration allows to reproject 3D points in true-scale.

In the case of a stereoscope with small baseline (order of the centimetre), most of the techniques use a 2D pattern with known dimensions and geometry.

Authors of [20] use a planar pattern to calibrate fisheye stereovision sensor.

In [21], Li et al. use an half-box 3D pattern to calibrate their fisheye stereoscope.

When the baseline between the two optical centres is large (order of the metre), and moreover when the field-of-view is large, as it is with our fisheye configuration (see Section 2.1), the dimensions of the pattern have to be huge and this solution is difficult to implement.

For BOSS project [23], the baseline between fisheye cameras is 1 m long, and a pattern with the shape of a cube of 1 m dimension is used.

Another solution is to place marks on buildings or known structures as in [36]. However, this answer needs to calibrate the system before every acquisition mission.

#### 1.5.2. Self-Calibration

Self-calibration methods do not rely on patterns. The principle is to automatically detect and match points of the scene in two pictures. Then, specific algorithms are able to estimate the pose between the two views and some of the internal parameters of the camera. These algorithms need a minimum set of points, as for example the 7-point and 8-point algorithms for standard cameras [37]. The problem of self-calibration is to find a robust way to choose good points pairs to apply the parameters estimation algorithm, to take into account the false positives in the detection and matching process. Generally a variant of RANSAC (RANdom SAmple Consensus) is adopted [38].

Self-calibration allows to calibrate the system without pattern of known dimensions. In this case, 3D reprojection can be computed at a scale factor and an additional reference measure is needed to find the real-scale of the scene.

For omnidirectional vision, the vision model is related to the sphere, and [24,25] propose two 9-point algorithms.

In [24], Mičušìk applies its algorithm to fisheye and catadioptric cameras, with simple projection functions at one (9 points couples are needed) or two parameters (in this case 15 points couples are needed).

In [25], Geyer and Stewenius apply their algorithm only to catadioptric sensors and propose a variant of 15 points in order to be able to estimate different internal parameters for two cameras.

In our case of stereoscope with large baseline and large field-of-view, a manual calibration is a constrained task.

In addition, for embedded sensors on a vehicle, it is shown in [39] that the sensors suffer from vehicle vibrations when moving and that the calibration can not be reliable within time. Hence a re-calibration should be necessary.

For all of these reasons we opt for a self-calibration algorithm inspired from [24] and expanded in [40]. The 9-point algorithm differs from 7 and 8-point algorithms in that it estimates not only the essential matrix but also a separate parameter of the function that represents the fisheye projection. An initial value of this parameter must be given before calibration, it is chosen from manufacturer technical characteristics of used lens (depends on fisheye projection and field of view).

With the 9-point algorithm, the essential matrix holds extrinsic parameters and the additional parameter is the intrinsic one. They are simultaneously estimated, but the intrinsic parameter is the same for both cameras. So, the theoretical accuracy is not as good as with a separate calibration of both lenses. But this method has the advantage to allow re-calibration during time to keep a quite good precision.

Essential matrix can be decomposed to extract the rotation between the two points of view, and the translation vector up to a scale. In presented stereovision configuration, the scale is recovered thanks to the knowledge of the baseline length between both cameras. Decomposition of the essential matrix is explained in [41].

### 1.6. Works on Matching for 3D Scene Reconstruction

To match the points between at least two images of different point of view is needed to achieve the 3D reconstruction. Matched points pairs allow to project corresponding rays, and, assuming neither sampling nor matching errors, lead to the 3D at their intersection.

#### 1.6.1. Sparse Matching Methods

Sparse matching methods take into account specific primitives in the images. They are robust, can work with rough calibration, but does not reach a full 3D modelling of the scene [42]. Different kinds of primitives can be studied:

- Corners [26]
- Boundaries [27]
- Curves [28]

For specified application, we aim at a detailed 3D model. For this reason, we will prefer a dense method.

#### 1.6.2. Dense Matching Methods

Dense matching methods find corresponding point for every pixel of the images. They reach more details, allowing more precision of the reconstruction. The counterparts are that resulting data are noisier and computation time is longer.

Many matching algorithms exist and exploit constraints to add robustness and reduce computation time.

Some are based on a sparse method, and try to densify matched points. Authors like [33] propose this way a belief propagation procedure.

Other methods measure the correlations from sliding windows, this is the case in [34].

More specifically, global methods regroups algorithms based on global optimization problems [42]:

- In [29], Lee et al. apply a simulated annealing approach;
- Authors of [30] propose a dynamic programming variant for stereo matching;
- Graph-cut methods allow robust results [31];
- Other global and slow methods are based on Hopfield neuronal network or genetic algorithms [32].

For the presented application, a dynamic programming approach has been chosen. Algorithm of Forstmann et al. [30] has been adapted to omnidirectional vision and geometry. This algorithm is a dense method, it matches every point of the images. Compared to state-of-the-art methods, it is a good compromise between execution time and accuracy. We use a variant with improved robustness to sampling in the case of fisheye stereovision [40].

### 1.7. Paper Presentation

To robustly find and process NLOS PR, authors often use embedded 3D models or similar data. The proposed method is used to generate 3D data from pictures taken on the fly. Chosen calibration method is fully automatic to deal with the large baseline of our fisheye stereovision configuration and to allow re-calibrations for robustness to vibrations. Selected stereo matching algorithm is based on the dynamic programming in order to get dense point clouds, with a good balance between complexity and accuracy. Added values of proposed approach are the avoidance of the dependence to recorded data, heavy and hard to keep up-to-date, and first of all the knowledge of receiver position. The difficult task of fixing the receiver position in the 3D model is not needed as it is already known.

In the proposed approach, a 3D model of the local environment is created and used both for the detection of NLOS satellites and the pseudorange delay estimation. The structures geometry is integrated in the workflow. Sensors for true-scale 3D reconstruction and GPS positioning are all on board the same vehicle and are working in the same time, allowing to locate the built model relatively to the true position of GPS receiver’s antenna in the local environment, as explained in the begin of Section 2 . Knowing the relative location between the model and the antenna is the main advantage of proposed approach above classical approaches based on recorded 3D model for which its relative position with the antenna is a challenge to fix. In Section 3 the well-centred 3D model allows to detect NLOS status of hidden satellites from antenna. Then in a second step, Section 3.2 , the model contains data on the street necessary for the PR delay estimation. This way, the proposed PR correction procedure should be more robust than those created from measures in specific environments.

An other category of procedures giving 3D data about the environment and aiming at localization is the SLAM (Simultaneous Localization And Mapping). SLAM is studied in [43,44] for omnidirectional vision. In this case the concern is not only a 3D restitution or a navigation solution, but both in the same problem. SLAM aims at locating the mobile relatively to the environment, and for that it includes modelling and cartography of the area. It can be the core of a visual and inertial navigation system, where different kinds of measures are exploited and fused for accurate positioning [45] : GNSS positioning, dead-reckoning, visual odometry, etc. By passing several times at the same place, both localization estimation and mapping are refined.

Even if with both methods a 3D map is generated and a localization is proceeded, presented application is distinct from SLAM:

- Generation of the 3D model is a step for the PR delay estimation and the GNSS fix computation, whereas SLAM computes and refine simultaneously local mapping and localization within a unique problem formulation;
- In presented works the localization concerns improvement of GPS accuracy, and not the visual-based positioning in the local mapping;
- GNSS positioning is the output of our procedure, and can be, as well as SLAM, an input among others for visual and inertial navigation;
- Hence, both methods can be combined.

The first part of this paper deals with the vision method employed in order to model the 3D environment in a point cloud. The steps of the procedure are detailed, from self-calibration to stereovision principle, and then the plane fitting in the 3D point cloud. These semantic informations are used in the second part, about a pseudorange delay estimation according to a model considering 1 to 3 reflections for NLOS satellite signals. It is based on the drawing of visibility masks for NLOS satellites identification, and street width and building height measurements. Finally, results with not post-processed positions show very good positioning improvement, from tens of metres errors to less than ten metres errors.

## 2. True-Scale 3D Modelling for the Proposed Method

Proposed solution to improve GNSS accuracy in semi-occluded environment is to correct NLOS pseudoranges delay. This needs a 3D model of the local area. Common issues of state-of-the-art solutions are:

- Data must be updated as often as possible.
- Re-positioning in the 3D model is difficult as the receiver is not well-localised in the true world.

Our aim is to go farther to solve the problem from an autonomous manner. Proposed method consists in automatically building the surrounding environment 3D model. This way, generated 3D model fits the real state of the area and receiver position in the 3D model is already known with high precision.

The 3D model is generated from pictures of the car vision sensor. It is centred on the local camera frame. Cameras and GPS receiver’s antenna are separated within a rigid transformation (see configuration in Figure 4 ). Thanks to this transformation, generated model can be centred on antenna’s frame. In other words, modelled obstacles are intrinsically and accurately located according to the antenna. This innovation avoids the localization problem of the receiver in a recorded model in GPS WGS84 frame, because in this case the only way to know the receiver’s position is to compute its (inaccurate) GPS position in WGS84 frame before the fusion with 3D data. To pass from local frame to ENU (East North Up) frame is allowed by using vertical and geographic North axes. Satellites can be positioned in the ENU frame centred on the receiver antenna following their azimuths and elevations. So, proposed method eliminates receiver positioning problem because the 3D model is built relatively to this position.

**Figure 2:**
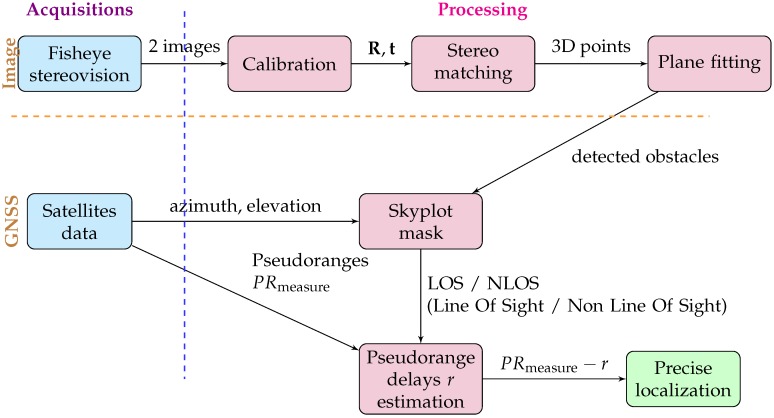
Procedure scheme for the 3D modelling and integration with GNSS (Global Navigation Satellite System) data. R,t = rotation and translation between cameras.

The specific methodology developed in this paper is shown in Figure 2. We consider a cheap configuration to acquire and compute 3D information, using two sky-facing fisheye camera. The algorithm is divided into 2 main parts: 3D modelling, and GPS improvement by integrating generated 3D data. Many steps appear in each of those parts. 3D modelling needs first a two views calibration step to know the relative orientation between positions and vehicle’s azimuth. Then, a complete matching process is applied to get a maximum of details of the area. The model is composed by a set of 3D points, within a plane detection can process in order to get semantic data of the scene. Planes are then used to generate a visibility mask in a skyplot. This mask is used to detect NLOS satellites. The correction of NLOS pseudoranges is based on the local geometry contained in the 3D model. The delay is then removed from NLOS pseudoranges and a more precise position can be computed.

### 2.1. Sample Scenes and Configuration

Two static scenes are presented and studied to show the effectiveness of the complete GPS improvement method:

- Cap3/0900, sequence duration = 29 s, Geographic North = 318°, before the junction between *rue Saint André* and *rue de la Halle*
- Cap4/1560, sequence duration = 32 s, Geographic North = 115°, before the junction between *rue Esquermoise* and *rue de Thiers*

**Figure 3:**
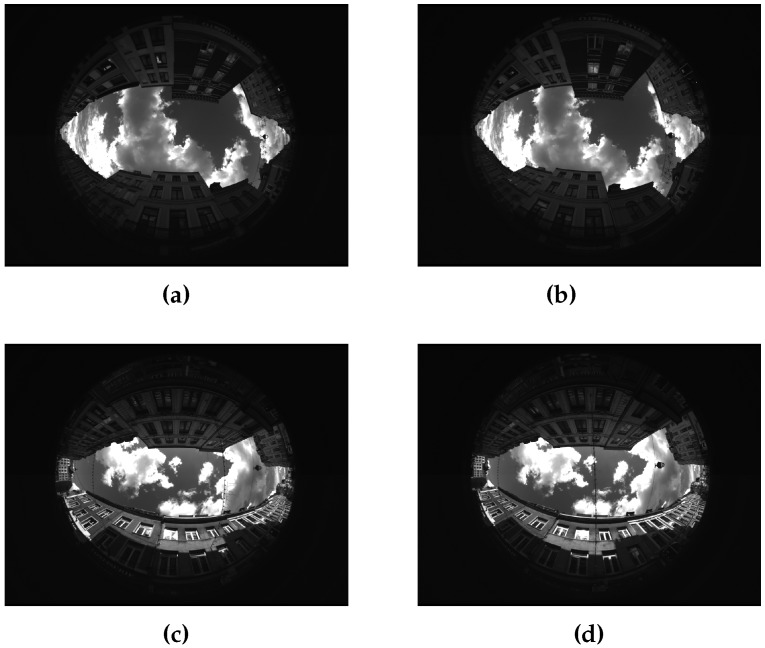
Fisheye views of presented scenes. (**a**,**b**) Cap3/0900, rear and front; (**c**,**d**) Cap4/1560, rear and front.

They have been taken in Lille, France, 19 August 2014. Figure 3 shows the images pairs of these two extracts.

**Figure 4:**
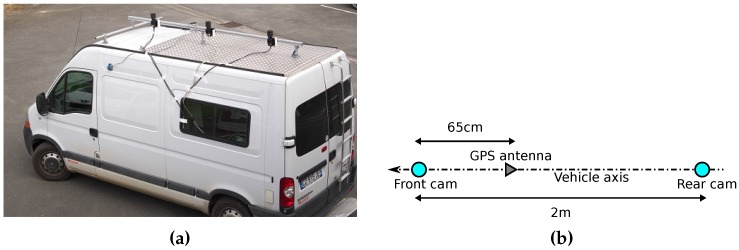
Truck with binocular stereovision configuration. The central camera of the picture is not used for this project. (**a**) IFSTTAR truck; (**b**) Sensors layout.

Experimental configuration is illustrated in Figure 4. The vision part is composed of two 8-bit greyscales JAI cameras (Copenhagen, Denmark) with Sigma 4.5 mm F2.8 fisheye lenses (Kanagawa, Japan) of 180° field of view in Nikon mount. Images are very high definition and the image circle has a diameter of 2310 pixels. Cameras are aligned along the vehicle longitudinal axis and the baseline is 2 m long. GNSS part is composed of a GPS receiver based on a lowcost u-blox chip with a patch antenna. This M3 Systems “Safedrive” receiver (Toulouse, France) is able to store raw data including pseudoranges. Neither Kalman filters nor other smoothing steps are applied to the data in order to compare raw positions before and after the pseudorange delay estimation and correction.

### 2.2. Fisheye Projection Model

The fisheye vision system observes the surrounding environment in two images. In order to not degrade data of fisheye images, we chose not to apply geometric distortions rectification that generates data by interpolation. 3D modelling is computed by following steps:

- Calibration is done to know intrinsic camera parameters and extrinsic parameters between two points of view;
- A matching step is applied according to stereovision principles and the epipolar geometry for spherical model cameras;
- The point cloud is processed to extract semantic information and to identify structures like walls.

**Figure 5:**
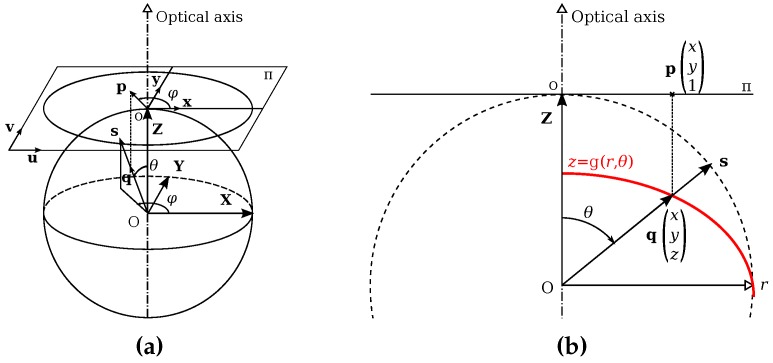
Model of omnidirectional fisheye projection with unique projection centre and symmetry around the optical axis. Π is the image sensor. (**a**) Unit sphere model; (**b**) Cross-section with the curve of a fisheye projection.

Fisheye projection is described by the unit sphere model as shown in Figure 5. This model is based on a projection function that describes the relation between the incident angle and the radius of the projected point on the sensor. Fisheye geometry can be represented by a profile with radial symmetry around the optical axis.

In Figure 5 the profile is represented by the g(r,θ) function. This curve allocates points projection on the sensor Π by giving the link between the incident angle *θ* and the distance *r* of projected point from optical centre. With the unit model, Π is tangent to the unit sphere following the optical axis. *r* and *θ* are dependant each other by the projection function r=proj(θ). If the function is reversible, getting *θ* from *r* can be done with $\theta ={\mathrm{proj}}^{-1}\left(r\right)$, and

(1) $$z=g\left(r,\theta \right)=\frac{r}{\tan(\theta )}$$

For presented works we use reversible fisheye functions. *z* can be written two ways:

(2) $$\begin{array}{cc} z & =g\left(\theta \right)=\frac{\mathrm{proj}(\theta )}{\tan(\theta )} \end{array}$$

(3) $$\begin{array}{cc} z & =g\left(r\right)=\frac{r}{\tan({\mathrm{proj}}^{-1}\left(r\right))} \end{array}$$

from these formulations, fisheye projection can be expressed the following manner.

From space to sensor plane:

*P* is a point observed by a fisheye camera. P is its corresponding vector in the frame centred on the camera optical centre. Vector P can be obtained by the following steps:

(4) $$P\left(\begin{array}{c} X\\ Y\\ Z \end{array}\right)\overset{÷∥P∥}{\to }s\left(\begin{array}{c} x_{s}\\ y_{s}\\ z_{s}=\cos\left(\theta \right) \end{array}\right)\overset{\arccos}{\to }\theta \overset{g(\theta )}{\to }z\to q\left(\begin{array}{c} x\\ y\\ z \end{array}\right)=s\cdot \frac{z}{z_{s}}\to p\left(\begin{array}{c} x\\ y\\ 1 \end{array}\right)$$

s is the projection of P on the unit sphere, with *θ* angle from optical axis. q is collinear to s and is in the same direction. They are identical with a sale factor. Knowing s we can get $\theta =\arccos(z_{s})$ and *z* by applying Equation (2). Relationship from s to q is given by $q=s\frac{z}{z_{s}}$. p is the orthogonal projection of q on Π according to the optical axis collinear to Z.

From sensor plane to space:

Counter-projecting a point p of the sensor Π gives an half-line from optical centre *O* following the unitary orientation vector s. It represents every space position projecting into p on the sensor.

(5) $$p\left(\begin{array}{c} x\\ y\\ 1 \end{array}\right)\to r=\sqrt{x^{2}+y^{2}}\overset{g(r)}{\to }q\left(\begin{array}{c} x\\ y\\ z \end{array}\right)\overset{÷∥q∥}{\to }s\left(\begin{array}{c} x_{s}\\ y_{s}\\ z_{s} \end{array}\right)$$

Each point p of Π projects itself on the unit sphere retina forming the ray s. *o* is the projection of the optical centre *O* on Π. The radius of p from the centre *o* is *r*. With *r* we get *z* from Equation (3). This way we get the 3 elements of q, that only needs to be normed to obtain s.

According to used projection model, parameters of function r=proj(θ) differ. Complex models have more parameters. For presented works we use the equisolid angle model, but we show in [40] that the same principles are valid with any of simple linear models.

### 2.3. Self-Calibration

As shown in Section 1.5, a self-calibration process is necessary to keep a good accuracy after decalibrations due to the vehicle’s vibrations, and is a good solution to calibrate the large-baseline and large-field-of-view (fisheye) stereoscope.

The used self-calibration process is based on the 9-point algorithm explained in details in [40]. It needs the automatic detection and matching between feature points from two images. It allows for estimating the intrinsic parameters of the camera as well as the pose estimation between the two points of view. SIFT feature points and descriptors are used (Scale-Invariant Feature Transform) [46] for their robustness to orientation and scale changes. Pose estimation is contained in the essential matrix, estimated by the 9-point algorithm. Rotation and translation between the two poses can be extracted from this matrix. Our calibration method allows to do a visual gyroscopy to follow the evolution of the vehicle azimuth. It is needed to project satellites in the images and in visibility masks.

**Figure 6:**
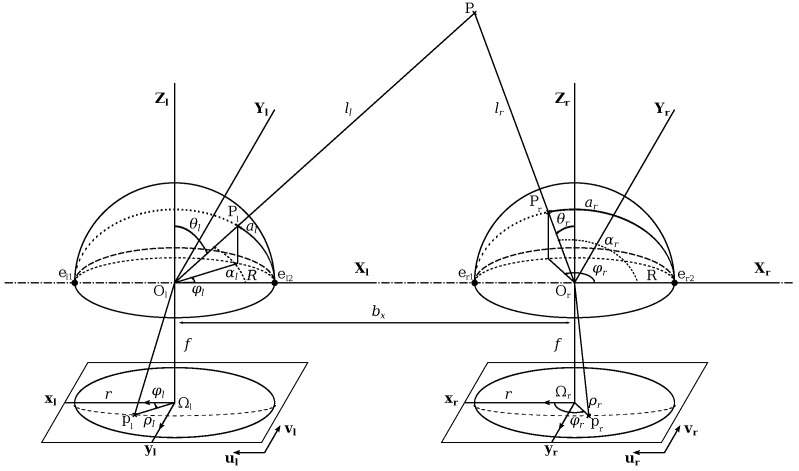
A stereoscopic sensor. Point *P* projects on the left and right spheres respectively in $P_{l}$ et $P_{r}$, and is seen as $p_{l}$ and $p_{r}$ in the images.

Figure 6 illustrates spherical epipolar geometry. With the spherical model, cameras have two epipoles $e_{1}$ and $e_{2}$, instead of a unique epipole with the usual pinhole camera model.

In the configuration Figure 6, points $e_{l1}$, $O_{l}$, $e_{l2}$, $e_{r1}$, $O_{r}$ and $e_{r2}$ are aligned and shape the epipolar axis. Epipolar curves are defined as the intersections of the plane $\left(O_{l},P,O_{r}\right)$ with the spheres describing fisheye lenses. Both epipolar curves are projected on the sensors following a fisheye projection function. As for the pinhole model, the calibration allows:

- to estimate model parameters linking every scene point *P* to its projection *p* in the image;
- to find parameters needed for the computation of conjugate epipolar curves from which the matching process is assessed.

**Figure 7:**
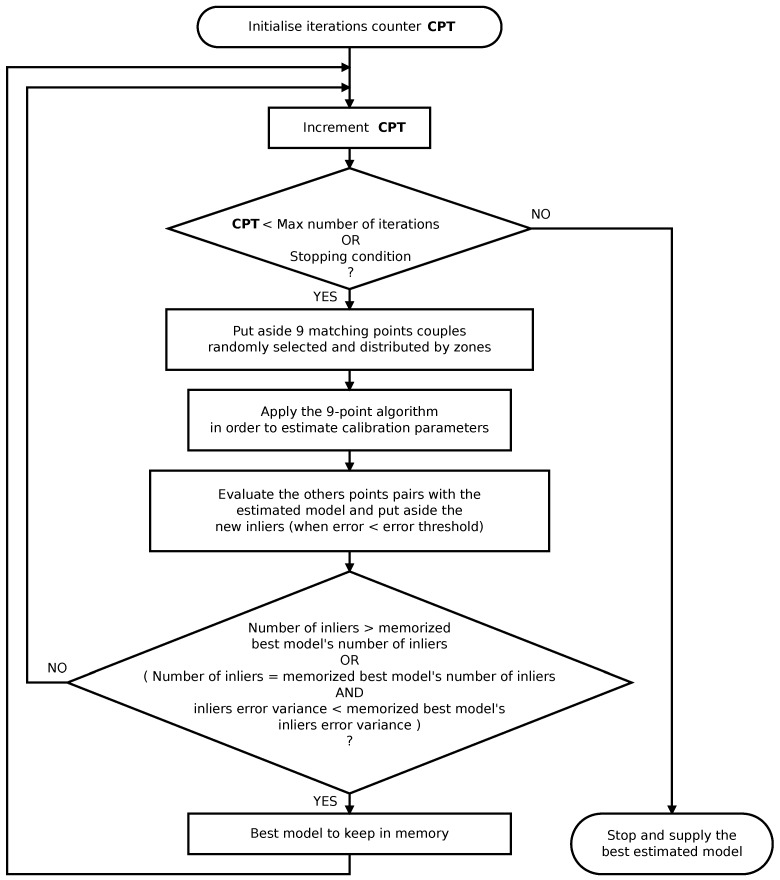
Pseudo-algorithm of the RANSAC (RANdom SAmple Consensus) strategy containing the 9-point algorithm.

A variant of the RANSAC loop masters the self-calibration [38]. Steps of the RANSAC-based calibration are presented in the pseudo-algorithm Figure 7.

Possible solutions for the projection function parameter and the essential matrix are estimated from 9 chosen points and an approximate initial value of the parameter.

**Figure 8:**
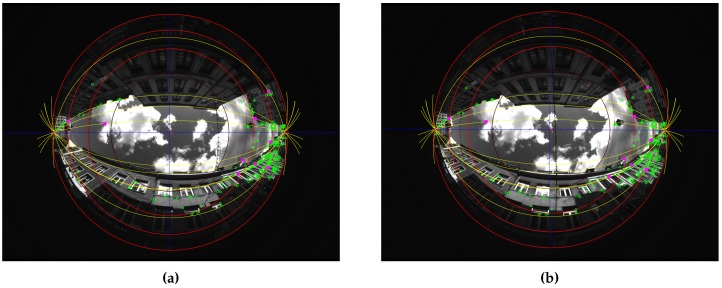
Illustration of the calibration, scene Cap4/1560 of acquisitions done in Lille. (**a**) Rear view; (**b**) Front view.

Figure 8 shows a calibration result for one of the presented scenes. Green points are the RANSAC inliers after the calibration process. Pink points are the 9 points couples giving the best model estimation according to the error measure. Yellow epipolar conjugate curves are those got from the pink points. They cross themselves in the two opposites epipoles.

### 2.4. Stereo Matching and 3D Scene Generation

Thanks to calibration, matching process is based on epipolar constraint: every point of an epipolar curve in an image has its corresponding point in the conjugate epipolar curve in the other image. More explanation and how to find conjugated epipolar curves in fisheye images can be found in [47].

Many matching processes have been developed in the literature and are introduced in Section 1.6. For presented works we use a dynamic programming algorithm optimized for fisheye images and improved by the use of a 3D graph as explained in [40].

**Figure 9:**
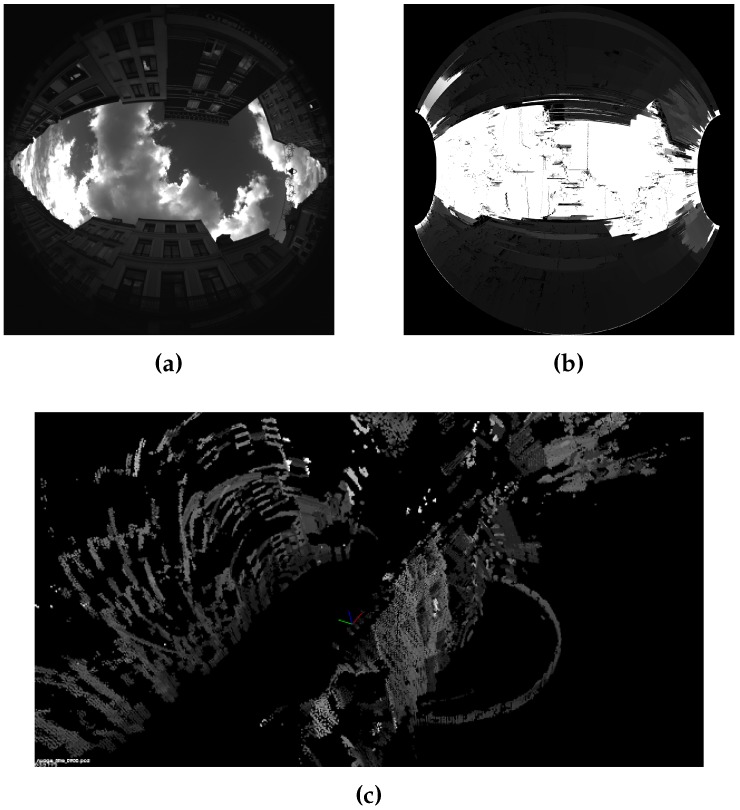
Distance map issued from the matching and resulting 3D model, scene Cap3/0900. (**a**) Initial rear image; (**b**) Distance map; (**c**) Filtered 3D point cloud.

**Figure 10:**
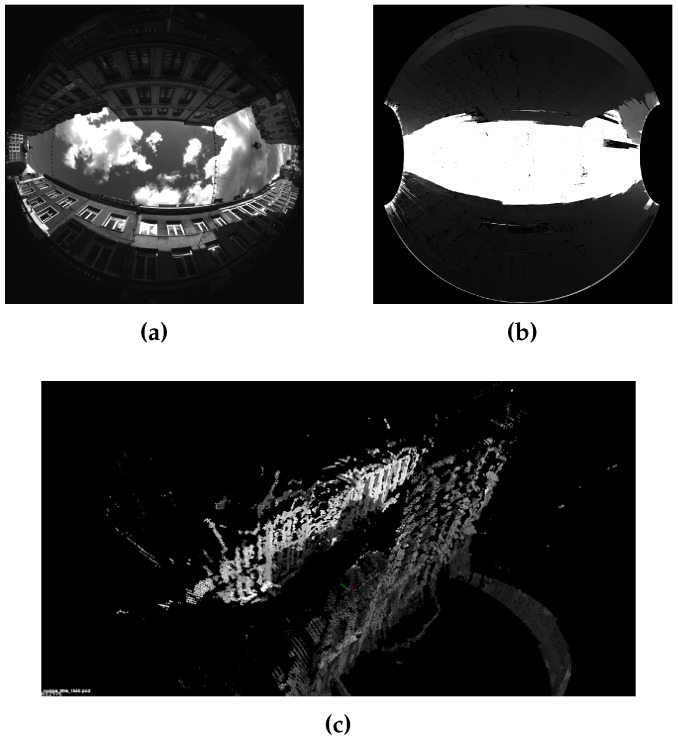
Distance map issued from the matching and resulting 3D model, scene Cap4/1540. (**a**) Initial rear image; (**b**) Distance map; (**c**) Filtered 3D point cloud.

Stereovision allows the computation of 3D points from the matching points in at least 2 images. Figure 6 illustrates this capability. 3D points computation is explained in [48]. The proposed method computes a 3D point cloud of the scene at real scale thanks to the knowledge of baseline length between the cameras. The obtained point cloud is dense, a filtering process is applied to reduce noise. With the method and parameters chosen in [40] we reach a metric accuracy. Figure 9 and Figure 10 show both sample scenes with distances maps and final 3D point cloud.

### 2.5. Plane Extraction and Evaluation

Selected plane extraction method is based on Hough transform in local ground maps generated from the 3D point cloud. This method is preferred over a RANSAC-based method that is more complex to implement to avoid false detections across the streets (see [40]).

From a 3D point cloud, it is possible to create 2D maps of the modelled area. Around a given position, we propose to create:

1. a 2D histogram in the XY plane, following the Z direction,
2. an elevation map.

**Figure 11:**
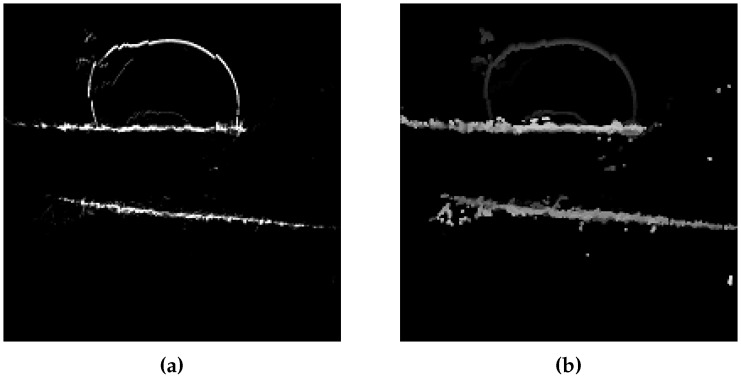
Ground imprints at the location Cap4/1560. Y axis is the bottom direction and X axis is the right direction. (**a**) XY histogram; (**b**) Heights map.

We sample the space according to a regular squared grid in XY. The histogram is computed by counting the number of 3D points in each region of the grid according to voxels. The dimension of the grid and voxels is 10 cm. The more a structure gets points following its height, the higher value the histogram’s box gets. In fact, the higher the points density is, the more we can be sure a structure exists here. The histogram is defined to have a maximal value of 255, that means it saturates for every structure that is at least 2 m above the GPS receiver’s level. For heights map, maximal *Z* is saved in an identical sampling grid. The heights map is filtered by a modified 3 × 3 median filter in order to smooth heights and reduce noise. The specificity of proposed filter is that it ignores negative or null values. Figure 11 shows an histogram (a) and associated heights map (b).

Maps are computed for bounds of 10 m in X and Y directions around reference position (receiver’s antenna). They have to be large enough to be able to detect complete data about the surrounding environment.

From ground prints we propose to exploit Hough transform to detect segments. They are the first elements used to define planes extracted in the 3D point cloud. Hough transform is explained in [49]. Advantages of Hough transform are that it can deal with detection of holes in the borders, and it has a good robustness to noise. It is used to detect straight lines or circles in black and white pictures. The probabilistic Hough transform is an extension allowing the detection of segments instead of lines [50]. This variant is used in presented application.

The map needs processing before detecting the segments: imprints have to be thin, with low noise, and binary. To do that, we apply following post-processing of the histogram in XY:

1. 1 pixel dilation and erosion (closing), to fill holes in the imprint;
2. Binary thresholding;
3. Thinning of Zhang and Suen [51] in order to avoid the detection of overlapping segments.

**Figure 12:**
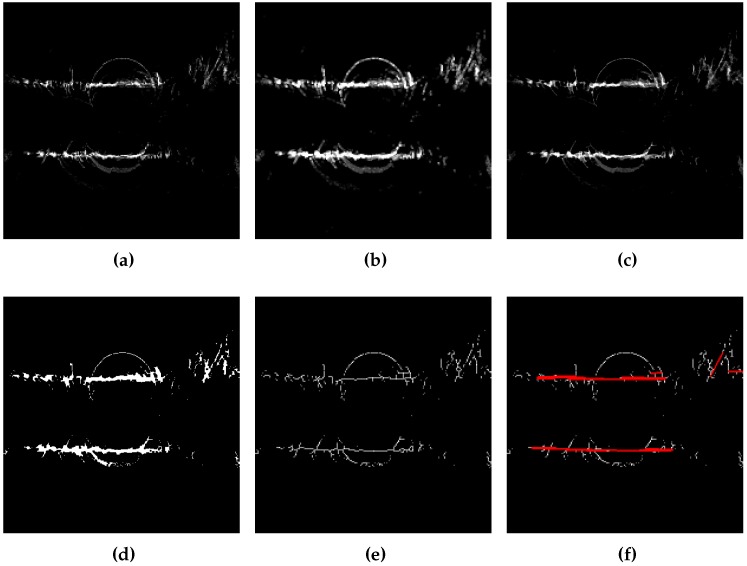
Processing applied to the computed ground map from the built 3D point cloud and segments detection by probabilistic Hough transform for the position Cap3/0900. (**a**) Ground print (XY histogram); (**b**) Dilatation; (**c**) Erosion; (**d**) Binary threshold; (**e**) Thinning of Zhang and Suen; (**f**) probabilistic Hough transform.

From these thin imprints we can detect segments with the probabilistic Hough transform. Figure 12 shows the steps for the segments detection from XY histogram.

After detecting the segments we can locate the structures in the 3D model. The missing information is the height of the walls. It is possible to define polygons from the segments and heights map as following: every structure begins from receiver’s level, and upper points are defined with the positions of segments ends and corresponding heights along Z from the heights map. This way we get a quadrilateral mesh. We apply the adaptive triangulation [52] in order to get a triangle mesh.

Evaluating the plane fitting is not an easy task without a precise 3D model of the environment. We propose criteria that shall fit well processed data for the presented application: a visual evaluation by reprojection of detected elements in the fisheye images allowing to see if heights are well-estimated, and a measure of the street width from cartography data. The reprojection is possible thanks to the projection model and the calibration done in Section 2.2 and Section 2.3. The qualitative criteria are:

- Ground prints and detected segments;
- Projection of top points in the fisheye view;
- Projection of extracted planes in the fisheye view.

The quantitative criterion is:

- Street width estimated from extracted planes (Width between the structures’ planes facing along the street).

Facing planes are not perfectly parallel. In addition, planes may overlap between them. In order to measure street width, and in order to fit the adopted PR reflection delay estimator in Section 3.2, we simplify it as a trench of constant width in the local model. Distance between each segment and street axis is measured perpendicularly. For both sides, the median measure is kept as the distance of this side from the trench centre. These two distances are cumulated to obtain the street width. Table 2 shows the results of street width measurement, detected segments and reprojection of higher points and detected planes. True widths are measured on cadastral plan.

Presented results confirm the metric accuracy we are able to get with proposed procedure. They are impressive considering the model is built from two fisheye images only, and with a matching algorithm that is not the more robust of the literature.

## 3. GNSS Positioning Improvement Based on Generated 3D Model

Improvement of GNSS localization is performed in three steps:

1. Detection of NLOS signals;
2. Estimation of NLOS pseudoranges delay;
3. Position computation replacing NLOS pseudoranges by corrected ones if available.

The 3D model is used for the two first steps. It allows generating a visibility mask around the antenna position. This mask is used to detect NLOS satellites. Then, instead of excluding NLOS satellites and to avoid loosing availability, we exploit a geometric pseudorange delay estimation. This method uses local structures and geometric data. It relies on a reflection model considering a punctual ray. Finally, pseudoranges are computed according to the local geometry of obstacles and should be accurate enough to improve PR estimation, and hence, the position estimation.

**Figure 13:**
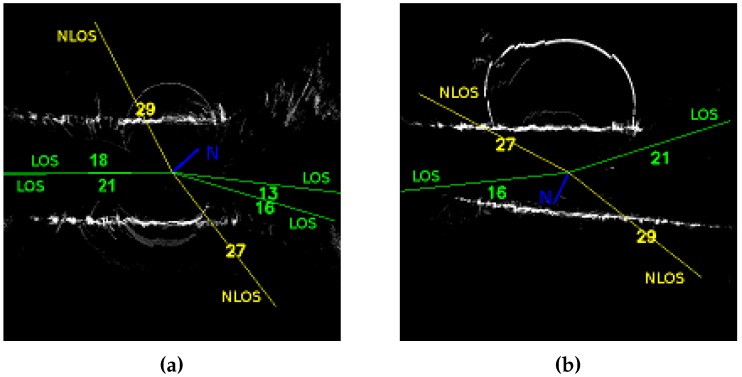
Ground prints of presented scenes. North orientation is plotted in blue. Green GPS rays are LOS and yellow ones are NLOS, according to the proposed mask of visibility. (**a**) Cap3/0900, satellites 18 and 21 are confounded in this representation as they have almost the same azimuth orientation.; (**b**) Cap4/1560.

Figure 13 shows ground marks around the two positions already presented in Section 2, with representation of hypothetical direct satellites rays. The centre position is the antenna position, and North orientation is plotted in blue. These positions are studied for the complete GPS localization improvement process, i.e. 3D modelling and pseudorange delay estimation. Experimental configuration is presented in Section 2.1.

### 3.1. Generation of Visibility Masks

**Figure 14:**
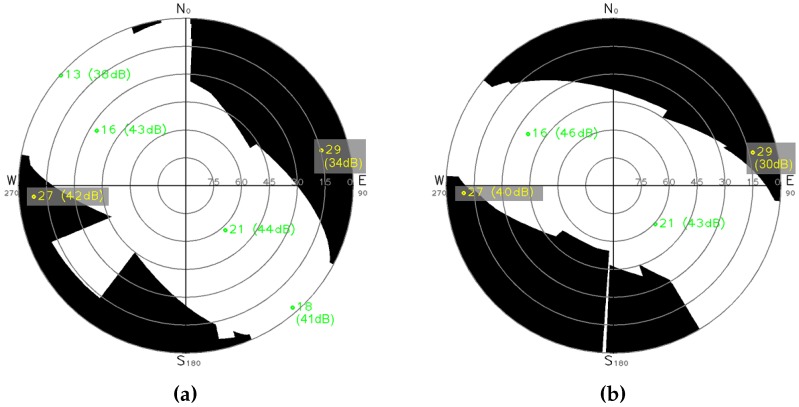
Skyplots and visibility masks for presented sequences. Green satellites are LOS and yellow ones bounded in grey are detected NLOS according to the mask. (**a**) Cap3/0900; (**b**) Cap4/1560.

Section 2.5 shows the extraction of surrounding buildings planes. These planes are used to generate the visibility mask in the skyplot at receiver’s position. The only additional information needed is the antenna position. This is already known because the acquisition system is rigid and relative positions between camera and GPS sensors are fixed. Projecting planes in the skyplot consists in a spherical projection, followed by an orthogonal projection onto the diagram. Figure 14 shows the skyplots obtained for sequences Cap3/0900 and Cap4/1560.

### 3.2. Delay Estimation with a 3 Reflections Model

Error model:

Reflections are the first source of pseudorange delays in canyons. So for now, we propose to estimate pseudorange delays of NLOS rays due to reflections only. Diffractions need more complex models to be treated. We call PR the pseudorange. For a NLOS signal, we write:

(6) $$PR_{\mathrm{corrected}}=PR_{\mathrm{measure}}-r$$

*r* is the pseudorange delay. These lengths are given in metres.

Reflection model for one to three reflections:

Planes are detected in the 3D point cloud. These planes represent the structures surrounding the vehicle. To simplify, studied model considered planes are parallels. They are used to measure the width of the street at the receiver position, the way is described in Section 2.5.

**Figure 15:**
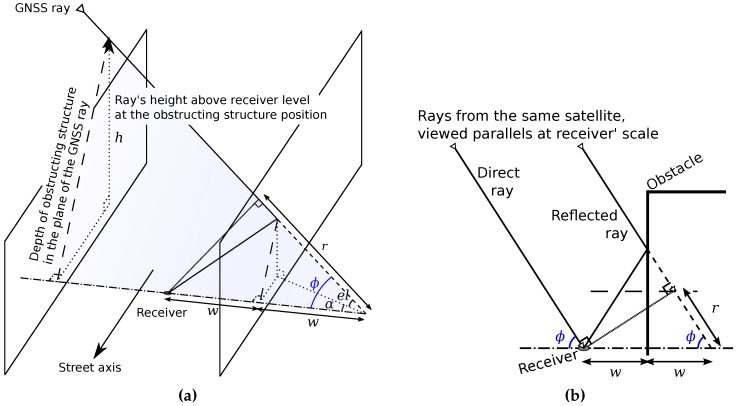
GNSS ray trajectory in the case of one reflection before reaching the receiver. (**a**) 3D illustration; (**b**) Cross-section following the plane of the GNSS ray and receiver.

Figure 15 shows the case of one reflection. The reflected ray meets the opposite wall of the blocking one. *w* is the distance between this wall and the receiver along its perpendicular angle. Delay *r* is given by:

(7) $$\begin{array}{cc} r & =2w\cdot \cos(\phi )\\ r & =2w\cdot \cos(el)\cos(\alpha ) \end{array}$$

*α* is the angle between the ray and the perpendicular axis to the reflection surface. This surface is supposed perfectly plane. el is satellite elevation, *α* is related to its azimuth and to the orientation of the perpendicular to the reflection plane and geographic North.

In order to improve the accuracy of the delay estimation, we follow the research of [19] to compute multiple reflections. Up to three reflections are estimated. In the case of more than three reflections occur, the signal becomes so weak that it is rarely perceived by the antenna. The idea presented in [19] is to consider a mask with several levels for the satellites. Each level corresponds to the number of reflections the ray shall have. In order to do that, Bétaille et al. simplify the model to get an “urban trench”, where the street is straight and infinite, and walls are verticals and parallels. Satellites with a lowest elevation meet more reflections.

**Figure 16:**
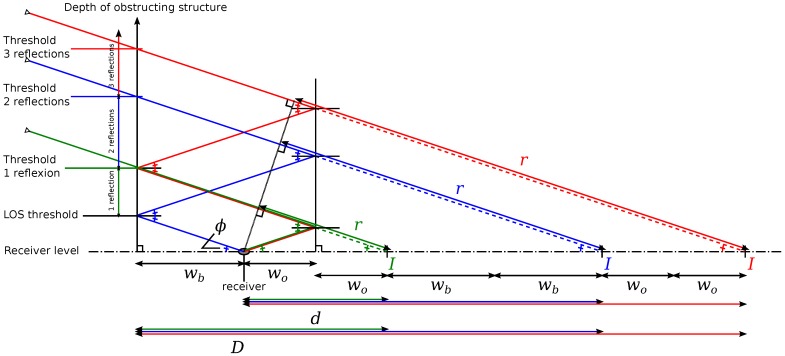
Reflected GNSS rays trajectories following the cross-section in the plane defined by the ray and receiver.

A NLOS ray has an elevation el and an azimuth az. *α* is the angle between az and the perpendicular axis to the reflection plane, $\alpha \in [0;\frac{\pi }{2}]$. A ray with multiple reflections is represented in Figure 16. $w_{b}$ is the distance between the wall blocking the NLOS ray and the street axis passing through the receiver, following the perpendicular axis of the wall. $w_{b}$ is the distance between opposite wall and the street axis passing through the receiver, following the perpendicular axis of the wall. Elevation thresholds, which define how much reflections the ray has and the corresponding delay, are formulated according to the limit height *h* of the blocking wall (Figure 15b). *d* is the distance between the receiver and the point *I* of the GNSS ray at the receiver level. *D* is the distance separating *I* from the blocking wall. *d* and *D* depend on $w_{b}$ and $w_{o}$, and $D=d+w_{b}$. For a ray at a given elevation, the height at the position of the blocking wall is defined by (Figure 15b):

(8) $$h=D\frac{\tan(el)}{\cos(\alpha )}\;\;\;\mathrm{and}\;\;\;el=\arctan\left(\frac{h}{D}\cos\left(\alpha \right)\right)$$

The delay due to the reflections is given by r=d·cos(ϕ). In addition cos(ϕ)=cos(el)cos(α). Detailed formulas are written in Table 3.

Extraction and evaluation of planes are detailed in Section 2.5. We can compare several ways of detecting LOS or NLOS state of a satellite. With the pseudorange delay rectification, we show in Section 4 that we compute a more accurate position.

In proposed works we detect the urban structures with a metric precision. Our street model is more precise than the “urban trench” to distinguish LOS and NLOS satellites, for example when neighbour buildings do not have the same height. In this case, the generation of a mask with several areas according to the number of reflections is complex to predict, the optimal method would be to estimate trajectories with ray-tracing from the receiver. However, in situations where the blocked ray has an azimuth close to street axis, the 3D model is not long enough to compute every ray-tracing with all the reflections.

That is why we finally propose the following solution: to use the visibility mask to identify NLOS satellites and to use the “urban trench” model to compute pseudoranges delays. Delay computation is done with NLOS satellites’ azimuth and elevation angles, and with 3D data: distance between opposite walls and axis street at receiver position and blocking structure’s height. This answer may not suits well the reality when the street is not straight. Our aim is to show the effectiveness of a pseudorange rectification from the generated 3D model from fisheye stereovision.

Proposed complete procedure is the following:

1. To extract the planes from the 3D point cloud and generate a visibility mask at receiver’s position;
2. To detect LOS and NLOS satellites with the visibility mask;
3. To measure structures distances on both street sides according to the “urban trench” model;
4. For NLOS satellites, to measure the height of blocking structure and estimate the delay *r* in order to compute $PR_{\mathrm{corrected}}$.

## 4. Evaluation of GPS Localization with Proposed Method

### 4.1. Validation Procedure

Used configuration does not embed true positions with precise enough synchronisation. In order to validate proposed method, we work on fixed positions to avoid synchronisation problems and get ground truth manually. True positions are not used in the 3D modelling and PR delay estimation workflow. True positions are only used to evaluate GPS positioning results. Manual truth does not include altitude, so 3D positions are not evaluated, the evaluation concerns 2D positions.

To show the effectiveness of our method, we propose to compare NLOS detection according proposed visibility mask with NLOS detection by CN0 threshold. To do that we need to look for an optimal CN0 threshold to distinguish NLOS signals. Authors like [2,3] used different configurations. CN0 depends on the type of receiver, antenna and external conditions, an unique optimal threshold does not exist for every situation. They find optimal CN0 threshold for their configurations respectively equal to 40 and 42 dBHz. For this reason, we propose to compare a CN0 threshold for each CN0 value between 40 and 42 dBHz, included.

### 4.2. Metrics

For the evaluation of 2D positions before and after correction, we use the following quantitative metrics:

- availability rate for 4 satellites (in percent);
- average error (euclidean distance) compared to the truth position (in metres);
- standard deviation (in metres).

We compute the metrics for the following corrections:

- NLOS exclusion;
- delay estimation for 1 reflection;
- delay estimation for 1 to 3 reflections,

These corrections come with each of the following NLOS detection methods:

- CN0 threshold: let thresh be the threshold value, is assumed NLOS a satellite whose CN0 < thresh otherwise it is LOS, to do for thresh∈[40;42] dBHz;
- proposed visibility mask obtained thanks to the generated 3D model.

### 4.3. Results

Table 4 shows the results of both sample scenes.

**Figure 17:**
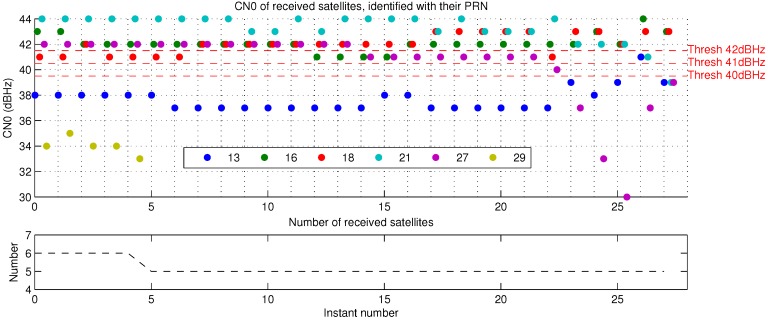
Satellites CN0 for sequence Cap3/0900.

NLOS rejection never gives good results. Availability decreases and is too low even with a short acceptance tolerance, especially with higher CN0 threshold. Above all, error dramatically increases whatever the NLOS detection method. However, in the case of Cap3/0900, Table 4a, error and standard deviation are lower with NLOS detection based on CN0 thresholds than on proposed visibility mask. Superior availability given by visibility mask indicates that it finds less NLOS satellites. In fact, only satellites PRN (PseudoRandom Noise number) 29 and 27 are shown NLOS by the visibility mask, during the complete sequence. Both satellites are projected in the mask Figure 14a (note that signal PRN 29 becomes unreachable from instant 5, see Figure 17). Figure 17 shows that satellite PRN 27 is not rejected by CN0 thresholds for more than half of the instants. We can explain lower average errors with CN0 thresholds for this record by a better geometric repartition of supposed LOS satellites used for position estimation. In Figure 14a, we can see that visibility mask keeps 4 LOS satellites, and most importantly that they are distributed along an almost straight line. If we consider satellite PRN 27 as LOS, even if one of the other satellites is rejected, horizontal geometric repartition is better and allows more precise localization. This geometric distribution is linked to the DOP measure. Author of [53] gives more details about DOP and how it is computed. HDOP (Horizontal Dilution Of Precision) and GDOP (Geometric Dilution Of Precision) are respectively defined to express DOP for horizontal satellites coordinates only, and to take into account satellites clock offset and complete positions (vertical and horizontal coordinates). The lowest the DOP is, the better the satellites repartition is, and the more accurate can be the GPS position estimation. Average GDOP with NLOS rejection according to the visibility mask for every instant is equal to 12.7362 (HDOP of 11.1328). Average GDOPs with NLOS rejection according to CN0 thresholds are equal to: 5.7016 for 42 dBHz (HDOP of 4.8466), 6.0132 for 41 dBHz (HDOP of 5.1254) and 6.0017 for 40 dBHz (HDOP of 5.1151). So, in this particular case, CN0 threshold NLOS detection seems to allow less errors than proposed visibility mask. Anyway, this is due to the better DOPs with NLOS rejection and the final result can not be considered better because of the service availability fall.

For pseudoranges corrections based on 1 or multiple reflections, the best results are those obtained with the NLOS detection based on proposed skyplot visibility mask. This is true for the error and the standard deviation, meaning it allows more accuracy and stability. NLOS distinction based on CN0 threshold can result in corrections with better accuracy than without corrections, but with less stable positions. Correction with 1 to 3 reflections gives better results than with only 1 reflection, excepted with scene Cap3/0900 and CN0 threshold NLOS distinction method.

What can we add concerning CN0 threshold NLOS detection?

**Figure 18:**
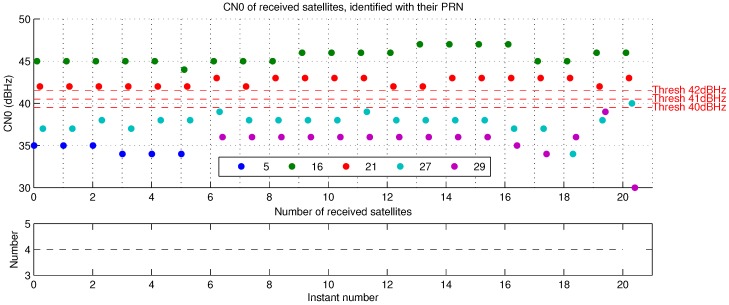
Satellites CN0 for sequence Cap4/1560.

Firstly, it is hard to choose a good threshold. With presented configuration, best CN0 threshold seems to be 42 dBHz. It gives the best results among all CN0 thresholds for both scenes (here we ignore the availability score as NLOS rejection has proven not to be a viable option). Figure 17 and Figure 18 show satellites CN0 at each instant for both presented scenes. The more the threshold is high, the more satellites are considered NLOS. For scene Cap3/0900 (Figure 17), CN0 of 42 dBHz detects more NLOS satellites for most of the instants. For scene Cap4/1560 (Figure 18), the only NLOS detection variation is at the last instant, between thresholds of and above 40 dBHz. That means that the threshold must be accurately chosen, a threshold of 1 dBHz more or less may lead to completely different and incoherent results.

**Figure 19:**
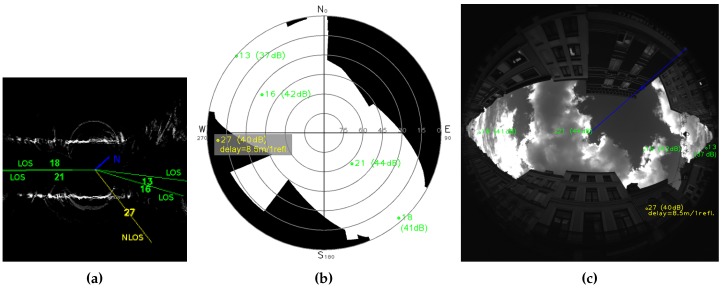
Instant 22 of acquisition Cap3/0900, pictures with satellites status given by the visibility mask method. The blue line indicates geographic North. LOS receptions are in green. NLOS receptions are in yellow with delay estimation. (**a**) Ground map with direct rays; (**b**) Skyplot and visibility mask; (**c**) Fisheye picture with satellites.

**Figure 20:**
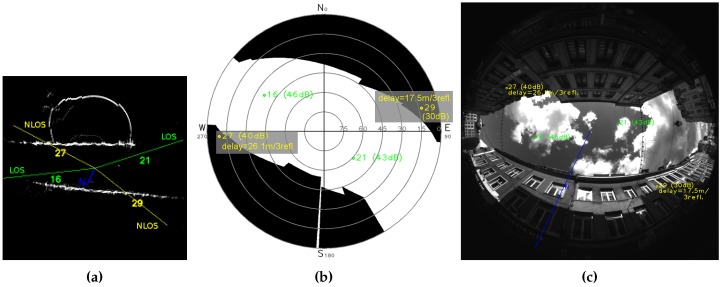
Instant 20 of acquisition Cap4/1560, pictures with satellites status given by the visibility mask method. The blue line indicates geographic North. LOS receptions are in green. NLOS receptions are in yellow with delay estimation. (**a**) Ground map with direct rays; (**b**) Skyplot and visibility mask; (**c**) Fisheye picture with satellites.

Secondly, CN0 threshold gives worse results after corrections than the visibility mask method. Corrections methods are the same, so, we can imagine CN0 threshold achieves worse NLOS detection than the skyplot mask. Figure 19 and Figure 20 show the efficiency of visibility mask method, as a check of the NLOS detection is possible according to the reprojection fisheye image and ground map (see Section 2.5) with direct GPS rays. CN0-based NLOS detection is as good as the visibility mask for scene Cap4/1560 (Table 4b), from 41 dBHz. Results are identical, meaning NLOS detection is the same for both methods. CN0 threshold of 40 dBHz gives worse results, it detects less NLOS, and it surely fails: Figure 18 shows there is only one difference, at instant 20 for the satellite PRN 27. At this time, as illustrates reprojection pictures of Figure 20, satellite PRN 27 is projected on the buildings and the visibility mask, it must be NLOS like for others times. This difference of NLOS detection is an error and shows that CN0 is variable and consequently a fixed CN0 threshold is not reliable. For scene Cap3/0900 (Table 4a), results based on CN0 threshold never reach results with skyplot visibility mask, and are sometimes worse than without any pseudorange correction. We propose to focus on instant 22, where every CN0 threshold give different results. Figure 19 shows that NLOS satellites must contain only satellite PRN 27 (40 dBHz). Doubt is allowed for satellite PRN 13 (37 dBHz at instant 22), because of its generally low CN0 (Figure 17: from 37 to 39 dBHz, and only once 41 dBHz). For CN0 thresholds of:

- 40 dBHz, only satellite PRN 13 is detected NLOS;
- 41 dBHz, satellites PRN 13 and 27 are detected NLOS, it might be the best CN0 threshold result for this instant;
- 42 dBHz, satellites PRN 13, 27 and 18 (41 dBHz) are detected NLOS, detection of PRN 18 is a false detection as can be shown thanks to Figure 19.

All of these results show that the CN0 threshold is not a reliable way to detect NLOS signals because it must be precisely and well chosen, and because sometimes CN0 may not be representative of the LOS or NLOS status as it may have many variations (see PRN 13 and 27 in Figure 17). In this case, correction results are unpredictable and unstable. The proposed skyplot visibility mask gives the best correction results. It seems to be a reliable way to detect NLOS satellites. In addition, a reflection model with more than 1 reflection is the best of presented pseudorange delay estimations.

A combination of NLOS distinction with proposed visibility mask and multiple reflections delay estimation is the best choice among presented options. These results are promising, inaccuracy is reduced at least by 50% and error standard deviation at least by 30%. Our procedure ensures an improvement of GPS localization based on the local environment modelling.

## 5. Conclusions

This paper is devoted to the use of a locally generated 3D model in order to increase GNSS localization accuracy for the vehicle. We describe a method that generates the 3D point cloud on-line via calibration and matching steps. Buildings fronts are extracted and their positions are estimated from the 3D model. The modelling is centred on a local frame, allowing to locate structures relatively to the GPS receiver antenna as they are in real world. Thanks to that, we succeed in creating visibility masks in the skyplot around the vehicle from which we estimate accurately satellites states (LOS/NLOS) and pseudorange errors when satellites signals are reflected.

We are able to improve vehicle GPS position by correcting pseudoranges that have been prolonged after at least one reflection. We show good results with this correction for a static vehicle. Exploited model for one, two or three reflections is powerful. An extention would be to complete it with a diffraction model similar to the one presented in [18] in order to correct satellites whose masking is ambiguous in the borders between visible and masked areas.

A new masking method at receiver’s position is presented to find NLOS satellites. The mask is generated from the 3D model dynamically built. Such a mask is more accurate than a mask in the camera view as it takes into account the position bias between the GPS antenna and reference camera. This bias can disallow the distinction of ambiguous satellites if placed in the camera point-of-view. So, the mask built from extracted planes is more robust than a mask based on an image segmentation such as in [4].

In addition to the localization improvement, the advantages of proposed solution are:

- A correction instead of a rejection of NLOS, does not imply loss of service availability;
- No environment data to store;
- No updates of embedded data to do, because the structure of the environment is updated on line;
- No re-positioning problem in the 3D model, because the reference position is known thanks to the 3D model built around it.

However results show an improvement of availability and better and more stable results with NLOS detection by proposed visibility mask than with CN0 thresholds. GPS localization is more accurate thanks to the PR delay estimation based on generated 3D model of the environment.

In order to estimate PR delay due to reflections, a 3D model is needed. Our method allows using a 3D model without knowledge of the area, well-centred to robustly detect NLOS receptions and estimate PR delays. For this reason it can be used in every situation and is the first step to autonomous geometric PR delay estimation that outperforms every method that needs additional knowledge.

### 5.1. Perspectives

We show in [40] that the same procedure is as efficient with a single fisheye camera and odometer configuration. With this second configuration, mobile experiments also have been proposed. A GPS RTK (Real Time Kinematic) was added to get a ground truth for the path. Pictures were taken every 2 m thanks to the odometer, and they were timed with an accuracy of one second only (the camera of this platform does not store more precise clocks). Even by applying time offsets, dynamic results never matched static ones. The problem of synchronisation between GPS and image data is the challenge to solve for dynamic conditions. We think that without ultra-precise synchronisation (approx. hundredth or thousandth of second), the problem is too complex and a classic reflection model is inefficient.

We propose a successful procedure to correct pseudoranges delays due to local reflections. This way of research can be continued to increase results. Perspectives in mind are:

- To develop a real-time implementation of the procedure;
- To extend the reflection model to a reflection and diffraction model;
- To experiment with more than one GNSS constellation (currently only GPS);
- To use a configuration with very precise data synchronisation (≪ 1 ms) in order to evaluate the method with synchronised GPS RTK truth and in dynamic situations;
- To detect vegetation for a specific diffraction model;
- To improve the plane extraction to be more accurate in height boundaries.

The method has been tested in urban environments, we can imagine using it in other specific semi-occluded environments.

## Figures and Tables

**Table 1:** Related works summary. Bold topics represent selected solutions for proposed method.

Topic	Sub-Topic	References
**NLOS detection**	CN0 threshold	[2,3,4]
	Vision segmentation	[5,6,7,8]
	Recorded 3D data	[9,10,11,12,13,14,15]
	**Online 3D data**	[16] (lidar)
Positioning considering LOS/NLOS status	NLOS rejection	[4,6,7,9,12,16]
	LOS/NLOS weighting	[5]
	LOS/NLOS Kalman filter	[10,11]
	Shadow matching	[13]
**PR delay correction**	**Local reflections**	[3,17,18,19]
	Local diffractions	[3,18]
	Multipath	[3,18]
	Delay simulation at candidates positions	[14,15]
	Estimator based on measures	[8]
**Omnidirectional vision calibration**	Pattern	[20,21,22,23]
	**Self-calibration**	[24,25]
**Stereo matching**	Sparse	[26,27,28]
	**Dense**	[29,30,31,32,33,34]

**Table:** (**a**)

Scene	Maps and Projections		
	2D Segments	Top Points	Planes
Cap3/0900	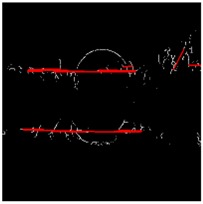	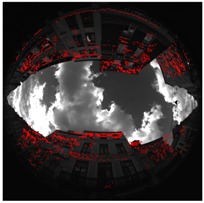	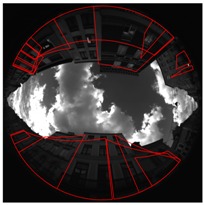
Cap4/1560	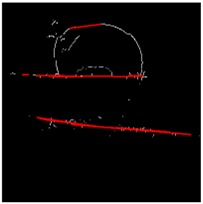	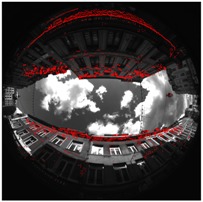	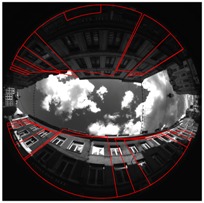

**Table:** (**b**)

Scene	True Width	Trench Measure	Difference (Accuracy)
Cap3/0900	12.3 m	12.35 m	0.05 m
Cap4/1560	9.5 m	10.52 m	1.02 m

**Table 3:** Table of NLOS pseudorange delay measurements due to reflections.

el⩽	Reflections	Delay r=d·cos(el)cos(α)
$\arctan\left(\frac{h}{2w_{o}+w_{b}}\cos\left(\alpha \right)\right)$	1	$2w_{o}\cdot \cos\left(el\right)\cos\left(\alpha \right)$
$\arctan\left(\frac{h}{2w_{o}+3w_{b}}\cos\left(\alpha \right)\right)$	2	$2\left(w_{o}+w_{b}\right)\cdot \cos\left(el\right)\cos\left(\alpha \right)$
∞	⩾ 3	$2\left(2w_{o}+w_{b}\right)\cdot \cos\left(el\right)\cos\left(\alpha \right)$

**Table:** (**a**)

Correction	LOS and NLOS Distinction Method			
	40 dBHz	CN0 Threshold 41 dBHz	42 dBHz	Proposed Visibility Mask
Without	2D positioning $\hat{\mathrm{error}}$ = 22.73 m; Standard deviation = 7.31 m			
NLOS rejection	Avail = 85.71%	Avail = 82.14%	Avail = 21.43%	Avail = 100%
	$\hat{\mathrm{Error}}$ = 61.77 m	$\hat{\mathrm{Error}}$ = 61.83 m	$\hat{\mathrm{Error}}$ = 63.35 m	$\hat{\mathrm{Error}}$ = 98.73 m
	Std dev = 3.21 m	Std dev = 3.27 m	Std dev = 2.51 m	Std dev = 17.78 m
1 reflection	$\hat{\mathrm{Error}}$ = 21.34 m	$\hat{\mathrm{Error}}$ = 20.84 m	$\hat{\mathrm{Error}}$ = 16.96 m	$\hat{\mathrm{Error}}$ = 12.64 m
	Std dev = 9.52 m	Std dev = 9.47 m	Std dev = 8.45 m	**Std dev = 1.80 m**
1 to 3 reflections	$\hat{\mathrm{Error}}$ = 22.69 m	$\hat{\mathrm{Error}}$ = 22.19 m	$\hat{\mathrm{Error}}$ = 18.60 m	**$\hat{\mathrm{Error}}$ = 11.50 m**
	Std dev = 11.59 m	Std dev = 11.49 m	Std dev = 10.19 m	Std dev = 3.13 m

**Table:** (**b**)

Correction	LOS and NLOS Distinction Method			
	40 dBHz	CN0 Threshold 41 dBHz	42 dBHz	Proposed Visibility Mask
Without	2D positioning $\hat{\mathrm{error}}$ = 16.70 m; Standard deviation = 3.63 m			
NLOS rejection	Avail = 0%	Avail = 0%	Avail = 0%	Avail = 0%
	$\hat{\mathrm{Error}}$ = N/A	$\hat{\mathrm{Error}}$ = N/A	$\hat{\mathrm{Error}}$ = N/A	$\hat{\mathrm{Error}}$ = N/A
	Std dev = N/A	Std dev = N/A	Std dev = N/A	Std dev = N/A
1 reflection	$\hat{\mathrm{Error}}$ = 12.85 m	$\hat{\mathrm{Error}}$ = 12.47 m	$\hat{\mathrm{Error}}$ = 12.47 m	$\hat{\mathrm{Error}}$ = 12.47 m
	Std dev = 4.65 m	Std dev = 3.58 m	Std dev = 3.58 m	Std dev = 3.58 m
1 to 3 reflections	$\hat{\mathrm{Error}}$ = 7.49 m	**$\hat{\mathrm{Error}}$ = 6.38 m**	**$\hat{\mathrm{Error}}$ = 6.38 m**	**$\hat{\mathrm{Error}}$ = 6.38 m**
	Std dev = 6.74 m	**Std dev = 2.20 m**	**Std dev = 2.20 m**	**Std dev = 2.20 m**

## Abbreviations

The following abbreviations are used in this manuscript:

CN0	Carrier-to-receiver Noise density
DGNSS	Differential Global Navigation Satellite System
DOP	Dilution Of Precision
EKF	Extended Kalman Filter
ENU	East North Up
GDOP	Geometrical Dilution Of Precision
GNSS	Global Navigation Satellite System
GPS	Global Positioning System
HDOP	Horizontal Dilution Of Precision
HMRF-EM	Hidden Markov Random Fields Expectation-Maximization
IMU	Inertial Measurement Unit
LOS	Line Of Sight
NLOS	Non Line Of Sight
PR	PseudoRange
PRN	PseudoRandom Noise number
RAIM FDE	Receiver Autonomous Integrity Monitoring, Fault Detection and Exclusion
RANSAC	RANdom SAmple Consensus
RMS	Root Mean Square
RTK	Real Time Kinematic
SIFT	Scale-Invariant Feature Transform
SLAM	Simultaneous Localization And Mapping
WGS84	World Geodetic System 1984
